# Medical insurance and health equity in health service utilization among the middle-aged and older adults in China: a quantile regression approach

**DOI:** 10.1186/s12913-020-05423-y

**Published:** 2020-06-17

**Authors:** Guorui Fan, Zhaohua Deng, Xiang Wu, Yang Wang

**Affiliations:** School of Medicine and Health Management, Tongji Medical College, Huazhong University of Science and Technology, 13 Hangkong Road, Wuhan, 430030 China

**Keywords:** Social basic medical insurance, Health equity, Utilization of health services, Quantile regression

## Abstract

**Background:**

China has achieved nearly universal coverage of the Social Basic Medical Insurance (SBMI), which aims to reduce the disease burden and improve the utilization of health services. We investigated the association between China’s health insurance schemes and health service utilization of middle-aged and older adults at different quantiles, and then explored whether the SBMI could help reduce the underutilization of health services among the middle-aged and older adults in China.

**Methods:**

Survey data of middle-aged and older adults were drawn from the China Health and Retirement Longitudinal Study (CHARLS). A linear quantile mixed regression model was utilized to provide a comprehensive understanding of the relationship between SBMI and health service utilization, which was measured by the total medical expenditure. We took the New Rural Cooperative Medical Scheme (NCMS) as the reference level and examined the associations of the Urban Employee Basic Medical Insurance (UEBMI) and the Urban Resident Basic Medical Insurance (URBMI) with health service utilization.

**Results:**

The quantile regression analysis revealed a significant positive association between URBMI and health service utilization at the 0.75 (β = 1.608, *p* < 0.01), 0.8 (β = 1.578, *p* < 0.01), 0.85 (β = 1.473, *p* < 0.01), 0.9 (β = 1.403, *p* < 0.01) and 0.95 (β = 1.152, *p* < 0.01) quantiles, and also a significant positive association between UEBMI and health service utilization at the 0.85 (β = 1.196, *p* < 0.01), 0.9 (β = 1.070, *p* < 0.01) and 0.95 (β = 0.736, *p* < 0.01) quantiles. Results showed that URBMI was significantly associated with an improvement in inpatient health service utilization of the middle-aged and older adults, and a significant positive association between UEBMI and inpatient health service utilization was observed at 0.1 (β = 0.559, *p* < 0.01), 0.25 (β = 0.420, *p* < 0.05), 0.5 (β = 0.352, *p* < 0.05), and 0.75 (β = 0.306, *p* < 0.05) quantiles.

**Conclusions:**

Inequity in health service utilization exists among the middle-aged and older adults across urban and rural Chinese areas, and it can be explained by the different reimbursement benefits of SBMI types.

## Background

China has launched a large set of reforms regarding health insurance and healthcare since the beginning of the twenty-first century. The Social Basic Medical Insurance (SBMI) system that integrates the urban and rural sectors has achieved nearly universal coverage in terms of participation rate. By the end of 2017, the SBMI had grown to cover over 97% of Chinese population [1]. However, considering the deductibles, copayments, and maximum allowable costs, it provides limited service provision and financial protection with reimbursement rates of range from 44 to 68% [2]. Therefore, the current SBMI system is in its early stage that can be characterized as “wide coverage, basic protection”. Previous literature showed inconsistent results on the relationship between the SBMI and health service utilization, i.e., some studies reported that medical insurance could help increase health service utilization and improve health outcomes [2–7], while some others yielded different outcomes [8, 9]. Consequently, whether the SBMI achieves equitable utilization of health services needs further investigation.

Currently, there are three programs in SBMI system: the Urban Employee Basic Medical Insurance (UEBMI) for the urban employed, the Urban Resident Basic Medical Insurance (URBMI) for all the non-UEBMI-eligible residents in the urban area, and the New Cooperative Medical Scheme (NCMS) for the rural population [10]. By the end of 2014, the UEBMI, URBMI, and NCMS covered 283.3, 314.5, and 736 million people at 2842.4, 1628.4, and 408 yuan annual premium cost per capita, respectively [11, 12]. However, the insurance based on residency and employment status provides unequal benefits, which may result in inequity in health service utilization and health outcomes.

Our study aims to examine whether different types of SBMI lead to unequal utilization of health services among the middle-aged and older adults via a quantile regression approach. We adopt this approach for two reasons. First, the dependent variable in our dataset is highly left-skewed distributed, which suggests the advantages of quantile regressions over ordinary least squares (OLS). Second, quantile regression techniques have been widely used to study inequity issues [2, 13–16]. In this study, quantile regressions allow for estimation of different impacts of medical insurance types along the whole conditional distribution of health service utilizations, while the most commonly used OLS models are more suitable for estimation of mean utilization of health services.

By using the China Health and Retirement Longitudinal Study (CHARLS) data, we conducted an empirical research to measure the health service utilization of the middle-aged and older adults in China. We used quantile regressions to provide a comprehensive understanding of the relationship between SBMI and health service utilization at different quantiles of the distribution of medical expenditure. Our study also explored whether the SBMI in China could help reduce the gap between the utilization of health services among the middle-aged and older adults.

### Medical insurance system and health equity

China has established a basic medical insurance system covering both urban and rural residents [1]. Specifically, the UEBMI is a mandatory insurance scheme for employees in urban areas with premiums contributed by both employees and employers, and it covers expenses incurred by outpatient/inpatient services and at designated pharmacies. Urban individuals not covered by UEBMI such as unemployed urban residents (e.g., students and children), self-employed individuals, and employees in informal sectors can voluntarily join the URBMI program which is jointly financed by the government and enrollees [10, 17]. Rural residents enroll voluntarily in the NCMS as families, financed by central and local governments as well as individual contributions [5, 18].

Started in 1998, the UEBMI schemes are managed by cities/municipalities and financed by premium contributions by employer’s payroll tax (6% of the employee’s salary) and employee’s wages (2% wages) [18]. The URBMI was launched in 2008 and it is implemented at the city level and subsidized by the central and city governments [10]. In 2003, the State Council began to reestablish the NCMS program which was first launched in several provinces [18, 19]. It is implemented at the county level and subsidized by the central and county governments [2].

Health equity is the absence of systematic disparities in health (or its major social determinants) between more and less advantaged social groups [20]. Health inequities do not only pertain to inequities in health determinants but also refer to access to required medical resources and maintenance of health outcomes, as well as other inequities that violate equity and human rights [21, 22]. After the new medical insurance reform, the high coverage rate of the SBMI among the middle-aged and older adults in China has brought opportunities to access medical resources. However, lower coverage for vulnerable groups may not meet their healthcare needs may exacerbate the health inequity. Therefore, the achievement of equity in health, particularly the utilization of health services, remains still a problem. We payed close attention on health equity and analyzed the impact of the SBMI on the utilization of health services among the middle-aged and older adults in China.

## Methods

### Data source

This study drew data from the 2011, 2013 and 2015 CHARLS datasets. The CHARLS is a longitudinal study of individuals over age 45 in China aiming to understand the socioeconomic determinants and consequences of aging. The baseline national wave of CHARLS was fielded in 2011 and included approximately 10,000 households and 17,500 individuals in 150 counties/districts and 450 villages/resident communities. Participants are met for a follow-up visit every 2 years. By the time of the nationwide follow-up visits in 2015, the samples had covered 23,000 respondents in a total of 12,400 households.

Our sample only included residents involved in one of the three types of the SBMI system in mainland China. Since the CHARLS is a panel dataset and the variables of geographic location and access to care are only available in 2011 and 2013 waves, our study drew geographic location and access to care from 2011 and 2013 waves and other variables from the 2015 CHARLS wave. These three waves could be merged using respondent ID and community ID. Because of the change of the sample sizes in three-waves survey and the fact that residents could only enroll in one kind of SBMI, we removed those respondent samples which had more than one kind of insurance, no province records or no access to care records. The final sample size was reduced to 13,087.

### Measures

The survey instrument featured questions about demographics, self-reported health, chronic condition, demand for inpatient services, functional status, provinces, access to care, health insurance information and health services utilization. Specific details of each measure are described as follows.

#### Demographics

Sociodemographic factors including gender, age, education (illiteracy and literacy), marital status and income, were used in our study. Marital status was presented with two categories, namely, married and others (partnered, separated, divorced, widowed, never married). Income was measured by the household after-tax income.

#### Self-reported health

For Self-reported health, respondents were asked to rate their health status. Responses were divided into five categories: poor, fair, good, very good and excellent.

#### Chronic condition (chronic)

Chronic condition was measured by aggregating the affirmative responses to questions concerning chronic condition by asking respondents if they had at least one of the 14 chronic diseases included in the study (yes or no). Chronic patients were coded as “1”, and non-chronic respondents were coded as “0”. However, the chronic conditions estimated results were not reported in detail.

#### Demand for inpatient services (demand)

Respondent’s demand for inpatient services was measured by the question: “In the past year, did a doctor suggest that you needed inpatient care, but you did not get hospitalized?” Answers to this question were coded with 1 = “yes” and 0 = “no”.

#### Functional status

Functional status was measured according to the activities of daily living (ADLs) disability (range 0–6) and instrumental activities of daily living (IADLs) (range 0–5) [23].

#### Geographic location (provinces)

Geographic location was measured by the province in which the respondent is located (including 28 provinces). The provinces estimated results were not reported in detail.

#### Access to care

Access to care was assessed by the number of medical facilities in the respondents’ village/community. Our study computed three proxy measures: number of hospitals (Hospitals: including general hospitals, specialized hospitals and Chinese medicine hospitals), number of community health care centers (Health centers: including community health care center and community health care medical post) and number of township/village clinics in the community/village (Clinics: including township health clinic and village medical post) [24].

#### Health insurance information

Respondents’ health insurance information was measured by a self-reported item. Respondents were asked if they have any kind of SBMI coverage, including the UEBMI, URBMI, or NCMS.

#### Health service utilization

Previous studies have examined health service utilization with different measures. Some studies simply used the frequency of health service utilization such as total outpatient or inpatient visits during a period of time to measure health service utilization [25, 26]. While some others used the costs of receiving health services within a certain period and weighted to costs to sum the resource intensity overall healthcare services [27]. In this study, we use the total cost of receiving health services to measure the health service utilization of the middle-aged and older adults.

Our study aims to deviate from the impact of the medical expenditure differences caused by different diseases, geographic locations and types of outpatient and inpatient services. Therefore, we further controlled five variables, namely, self-reported health, chronic condition, demand for inpatient services, different levels of hospital and geographic location. In this manner, the total medical expenditure can better measure the health service utilization of the middle-aged and older adults.

Health service utilization in our analysis reflect the medical expenditure of different utilization of health services. The health service utilization satisfies two conditions, namely, (a) medical expenditure of hospitalization in the past year preceding the survey date and (b) medical expenditure of outpatient visits in the last month preceding the survey date [2, 4, 5, 28]. We combined the results of the two surveys and computed their logarithm.

### Quantile regression

To measure the differences across levels within groups, this study used a quantile regression approach [29]. Quantile regression obtains a different quantile function by examining the dependent variable distribution of different quantiles between (0, 1). Thus, a trajectory of the conditional distribution is formed. The quantile regression model can be expressed as follows [13]:

$$ {Q}_{\theta}\left(\left.y\right|x\right)=x\hbox{'}\beta \left(\theta \right), $$

where θ is the independent health service utilization of the samples; *x* is the explanatory variable; and regression coefficient *β*(*θ*) represents the interpretation of the independent variable *y* at the quantile level of the dependent variable. The corresponding *β*(*θ*) can be obtained by minimizing *β* in the following formula [29–31]:

$$ {n}^{-1}\sum \limits_{i=1}^n{\rho}_{\theta}\left({y}_i-{x}_i\beta \right) $$

with

$$ {\rho}_{\theta}\left(\mu \right)=\left\{\begin{array}{cc}\theta \mu & when\;\mu \ge 0\\ {}\left(\theta -1\right)\mu & when\;\mu \le 0\end{array}\right. $$

Then, our research model can be expressed as follows:

$$ y{\left(\mathit{\log}(expenditure)\right)}^{\tau }={\beta_1}^{\tau}\times age+{\beta_2}^{\tau}\times gender+{\beta_3}^{\tau}\times edu+{\beta_4}^{\tau}\times srh+{\beta_5}^{\tau}\times \log (income)+{\beta_6}^{\tau}\times marital+{\beta_7}^{\tau}\times chronic+{\beta_8}^{\tau}\times functional\ status+{\beta_9}^{\tau}\times demand+{\beta_{10}}^{\tau}\times access\ to\ care+{\beta_{11}}^{\tau}\times insurance1+{\beta_{12}}^{\tau}\times insurance2+\mathrm{p} rovinces\times {\gamma}^{\tau }+{constant}^{\tau }+{error}^{\tau }. $$

In our research model, *β*^*τ*^ is the fixed-effects coefficients for the *τ* th quantile, provinces are the random effects independent variables, and *γ*^*τ*^ is the random-effects coefficients for the *τ* th quantile.

Given that the sample is from many provinces in China, our study used Linear Quantile Mixed Models (LQMM) to test our model. We used the R language tool lqmm package for linear quantile mixed regression analysis [32]. And we reported the result in Table 2.

## Results

### Descriptive statistics

**Fig. 1:**
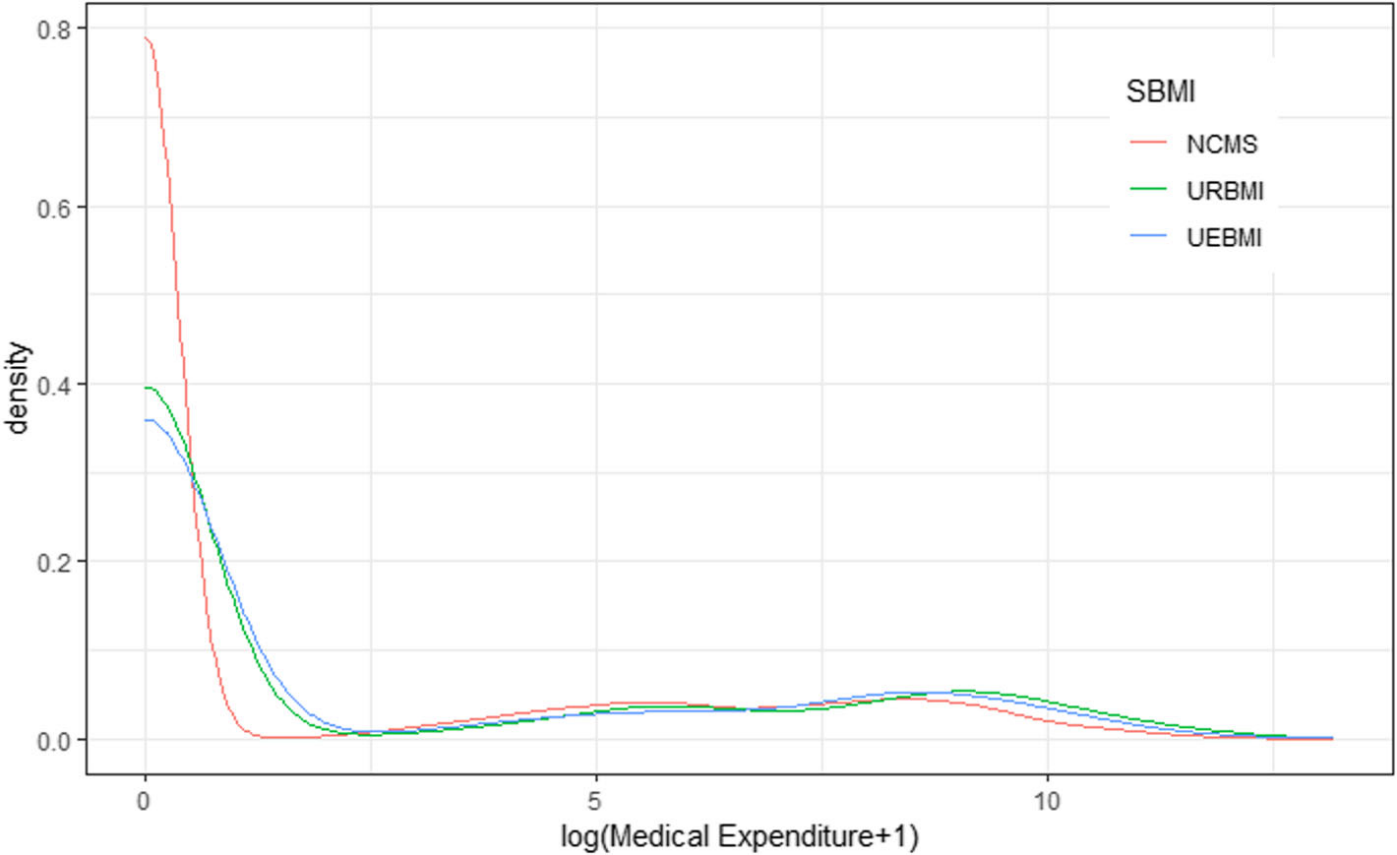
Medical expenditure distribution density

Figure 1 presented the medical expenditure distribution of the three programs of the SBMI. It showed that most medical expenditure of the three programs is 0. Given that the distribution of the dependent variable was highly-skewed, we chose the linear quantile mixed regression to fully understand the effect on the dependent variable at different quantiles. Considering that the dependent variable prior to the 0.75 quantile equals zero, our study only ran linear quantile mixed regression after 0.75 quantile.

Table 1 summarized the descriptive statistics of the variables. We reported the frequency and percentage for each categorical variable and the mean and standard deviation for each continuous variable. In the total sample, 83.2% (*n* = 10,886), 12.2% (*n* = 1596), and 4.6% (*n* = 605) were enrolled in NCMS, URBMI, and UEBMI, respectively. This result showed that NCMS covered the largest number of people in China. Almost half of the participants evaluated their health status as fair (54.9%), followed by poor (22.4%), good (11.3%), very good (10.2%), and excellent (1.2%). Based on the self-reported health information, we concluded that the majority of older adults were not in good health. The samples were divided into three groups according to the type of SBMI. Then we used the Kruskal–Wallis test to assess differences among the groups [33], and the results showed that there were substantial differences among the three groups. The descriptive statistics of Provinces and Chronic were shown in Table S1 (see Additional file 1).

**Table 1:** Descriptive statistics

	Total	NCMS	URBMI	UEBMI	Statistic^a^	*P*
	*n*(%)	*n*(%)	*n*(%)	*n*(%)		
*Demographic variables*						
Gender					127	< 0.001
Male	6247(47.7)	5060(46.5)	958(60.0)	229(37.9)		
Female	6840(52.3)	5826(53.5)	638(40.0)	376(62.1)		
Age					75	< 0.001
Mean ± SD	60.8 ± 9.3	60.5 ± 9.3	62.7 ± 9.5	61.4 ± 9.5		
Education					501	< 0.001
Illiteracy	3342(25.5)	3191(29.3)	73(4.6)	78(12.9)		
Literacy	9745(74.5)	7695(70.7)	1523(95.4)	527(87.1)		
Marital					17	< 0.001
Married	10,791(82.5)	8923(82.0)	1374(86.1)	494(81.7)		
Others	2296(17.5)	1963(18.0)	222(13.9)	111(18.3)		
Log(Income)					1515	< 0.001
Mean ± SD	4.5 ± 4.4	4 ± 4.1	7.8 ± 4.5	5.2 ± 4.7		
*DVs and other IVs*						
Self-reported health					118	< 0.001
Poor	2927(22.4)	2611(24.0)	199(12.5)	117(19.3)		
Fair	7191(54.9)	5927(54.4)	919(57.6)	345(57.0)		
Good	1476(11.3)	1145(10.5)	256(16.0)	75(12.4)		
Very good	1341(10.2)	1076(9.9)	203(12.7)	62(10.3)		
Excellent	152(1.2)	127(1.2)	19(1.2)	6(1.0)		
ADLs					46	< 0.001
Mean ± SD	0.4 ± 1	0.4 ± 1	0.2 ± 0.7	0.4 ± 1		
IADLs					179	< 0.001
Mean ± SD	0.4 ± 0.9	0.5 ± 1	0.2 ± 0.7	0.3 ± 0.8		
Demand					17	< 0.001
Yes	811(6.2)	706(6.5)	62(3.9)	43(7.1)		
No	12,276(93.8)	10,180(93.5)	1534(96.1)	562(92.9)		
Hospitals					344	< 0.001
One or more	8181(62.5)	6421(59.0)	1274(79.8)	486(80.3)		
No	4906(37.5)	4465(41.0)	322(20.2)	119(19.7)		
Health centers					1515	< 0.001
One or more	2396(18.3)	1350(12.4)	777(48.7)	269(44.5)		
No	10,691(81.7)	9536(87.6)	819(51.3)	336(55.5)		
Clinics					3291	< 0.001
One or more	10,669(81.5)	9821(90.2)	561(35.2)	287(47.4)		
No	2418(18.5)	1065(9.8)	1035(64.8)	318(52.6)		
Provinces^**b**^						
Chronic^**b**^						
Log (Medical Expenditure)					18	< 0.001
Mean ± SD	2 ± 3.4	2 ± 3.3	2.4 ± 3.8	2.2 ± 3.6		

Legends: ^a^: Kruskal-Wallis is used to assess differences between group

^b^: See Table 1S in Additional file 1

### Linear Quantile mixed models

To characterize the determinants of and differences in utilization of health services in various locations, our study performed linear mixed model (LMM) analyses followed by linear quantile mixed regression. We took NCMS as the reference level and examined the associations of URBMI and UEBMI with health service utilization.

For ease of illustration, Table 2 selected five representative quantiles, namely, 0.75, 0.8, 0.85, 0.9, and 0.95. In the case of controlling the demographic and control variables, a significant positive association between the URBMI and health service utilization at 0.75 (β = 1.608, *p* < 0.01), 0.8 (β = 1.578, *p* < 0.01), 0.85 (β = 1.473, *p* < 0.01), 0.9 (β = 1.403, *p* < 0.01) and 0.95 (β = 1.152, *p* < 0.01) quantiles was observed. These results demonstrated that URBMI was significantly associated with an improvement in health service utilization of the middle-aged and older adults, but at higher quantiles, the improvement in utilization of health services was smaller. Consequently, URBMI provided the most significant improvement in health service utilization for middle-aged and older adults with lower health service utilization. And in linear mixed model, URBMI was significantly positive associated with health service utilization for the middle-aged and older adults (β = 0.660, *p* < 0.01).

**Table 2:** Estimation results of health service utilization

Variables	LMM	Quantile				
		0.75	0.8	0.85	0.9	0.95
*Insurances*						
URBMI	0.660***	1.608***	1.578***	1.473***	1.403***	1.152***
	(0.100)	(0.388)	(0.287)	(0.238)	(0.224)	(0.158)
UEBMI	0.180	0.095	0.629	1.196***	1.070***	0.736***
	(0.140)	(0.258)	(0.397)	(0.382)	(0.239)	(0.220)
*Demographic variables*						
Age	0.009***	0.022***	0.034***	0.047***	0.055***	0.061***
	(0.003)	(0.007)	(0.009)	(0.009)	(0.008)	(0.011)
Gender	−0.058	−0.069	− 0.106	− 0.039	0.105	0.071
	(0.062)	(0.084)	(0.131)	(0.145)	(0.119)	(0.101)
Education	0.140*	0.278**	0.309*	0.297	0.237	0.388**
	(0.074)	(0.105)	(0.158)	(0.239)	(0.154)	(0.185)
Marital	0.041	0.085	0.218*	0.213	0.227	0.239**
	(0.075)	(0.093)	(0.118)	(0.130)	(0.168)	(0.110)
Log(Income)	−0.008	−0.003	− 0.017	− 0.020	− 0.051***	− 0.047****
	(0.007)	(0.008)	(0.016)	(0.018)	(0.018)	(0.013)
*Control variables*						
SRH						
*Fair*	−1.400***	−3.284***	−2.051***	−1.580***	−1.325***	−0.854***
	(0.076)	(0.337)	(0.297)	(0.159)	(0.111)	(0.092)
*Good*	−2.000***	−3.605***	−4.567***	− 3.841***	− 2.755***	− 1.565***
	(0.110)	(0.247)	(0.254)	(0.684)	(0.399)	(0.268)
*Very good*	−2.100***	−3.566***	−4.624***	−5.380***	− 3.310***	−1.812***
	(0.110)	(0.261)	(0.225)	(0.370)	(0.426)	(0.289)
*Excellent*	−2.100	−3.493***	−4.477***	−5.393***	−3.554***	− 2.712***
	(0.270)	(0.215)	(0.340)	(1.199)	(1.109)	(0.794)
Demand	1.400***	2.842***	1.978***	1.544***	1.025***	0.807***
	(0.120)	(0.342)	(0.193)	(0.284)	(0.216)	(0.188)
ADLs	0.170***	0.448***	0.331***	0.267***	0.257***	0.176***
	(0.036)	(0.121)	(0.101)	(0.090)	(0.060)	(0.059)
IADLs	0.021	0.124	0.175*	0.185**	0.047	0.082
	(0.038)	(0.090)	(0.088)	(0.079)	(0.058)	(0.049)
Hospitals	0.080	0.097	0.202	0.382**	0.378***	0.327**
	(0.062)	(0.083)	(0.121)	(0.153)	(0.121)	(0.136)
Health centers	−0.002	0.129	0.140	0.140	0.167	0.197
	(0.082)	(0.156)	(0.234)	(0.258)	(0.162)	(0.183)
Clinics	−0.064	0.132	0.104	0.331*	0.344	0.227
	(0.092)	(0.139)	(0.196)	(0.197)	(0.206)	(0.202)
Constant	2.100***	2.663***	2.998***	2.893***	4.000***	3.872***
	(0.260)	(0.413)	(0.583)	(0.665)	(0.563)	(0.603)
Observations	13,087	13,087	13,087	13,087	13,087	13,087
AIC	67,592	73,835	75,668	77,410	79,072	80,905
Log-likelihood	−33,764	−36,885	−37,802	−38,673	−39,504	−40,420

Notes

(a) Standardize coefficients are reported; standard errors in parentheses

(b) Significance level: ***p < 0.01, **p < 0.05, *p < 0.1

(c) We further controlled provinces and chronic conditions. Because the categories of variables were too many, the results are not reported in detail

Meanwhile, UEBMI was significantly positively associated with health service utilization at 0.85 (β = 1.196, *p* < 0.01), 0.9 (β = 1.070, *p* < 0.01) and 0.95 (β = 0.736, *p* < 0.01) quantiles. These results showed that UEBMI was associated with an improvement in health service utilization of the middle-aged and older adults at these quantiles, but this utility in health service utilization was smaller at higher quantiles. Accordingly, UEBMI had the most significant utility in health service utilization for middle-aged and older adults with middle-level health service utilization. Therefore, a certain degree of health inequity was observed between the NCMS coverage population and the URBMI and UEBMI coverage population. This inequity becomes evident at the urban and rural levels. However, no significant association between UEBMI and health service utilization was observed in linear mixed model.

### Impacts of the types of medical insurance on health service utilization

**Fig. 2:**
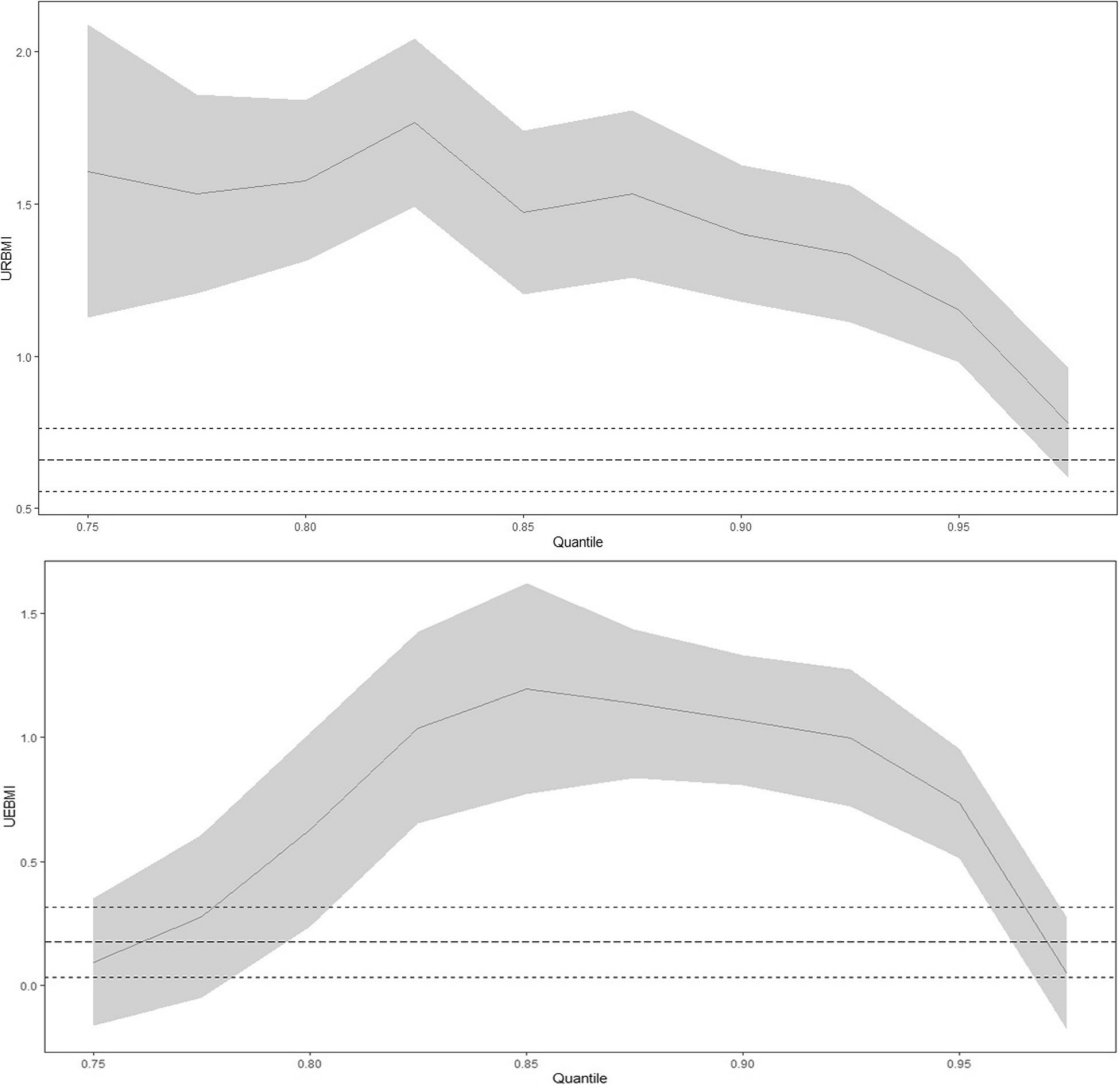
Linear quantile mixed regression results. The long-dashed line in the figure is the estimated value of the coefficient of each variable linear mixed model. The short-dashed line indicates the confidence interval of the linear mixed model estimation. The solid line represents the estimated value of the linear quantile mixed regression coefficient of each variable, and the shaded part refers to the confidence interval of the linear quantile mixed regression (linear mixed model and confidence interval of linear quantile mixed regression is 0.95)

In our study, the quantile coefficients at the 0.75–0.95 interval were calculated with a step-size of 0.025. Figure 2 presents a comprehensive picture of the linear mixed model results for important variables and the variation of coefficients with quantile in the linear quantile mixed regression. The figure presents the coefficients of URBMI on the utilization of health services, which fluctuated continuously between 0.75 and 0.80 quantiles and then increased to the peak at 0.83 quantile after which it could be witnessed a drop to the smallest coefficient at 0.97 quantile. This finding proved that a certain degree of inequity existed in the utilization of health services within URBMI.

Compared with NCMS, the coefficients of UEBMI were partly positive significant. Before 0.85 quantile, the coefficients of UEBMI gradually increased. And between 0.88 and 0.92 quantiles, the coefficients of UEBMI have fluctuated continuously. However, after 0.92 quantile, the coefficients of UEBMI decreased. This result proved not only the inequity utilization of health services among different types of SBMI but also the fact that a certain degree of inequity existed in the utilization of health services within a specific SBMI program. This finding also confirmed that a certain degree of health inequity was found between the middle-aged and older adults due to the different reimbursement benefits of the SBMI programs.

### Impacts of insurance on outpatient and inpatient health service utilization

Our study used the total medical expenditure to measure the health service utilization, and the outpatient medical expenditure may be largely different from the inpatient medical expenditure. For providing a comprehensive understanding of the relationship between SBMI and health service utilization, our study further divided research samples into three groups: middle-aged and older adults without any health service utilization (without any medical expenditure), middle-aged and older adults with only outpatient health service utilization (with only outpatient medical expenditure), and middle-aged and older adults with inpatient health service utilization (with or without outpatient medical expenditure). Our study also summarized the proportions of three groups in Table 3. From Table 3, the three different health service utilizations of NCMS account for 72.3, 15.2, and 12.5%, respectively. As for URBMI, the proportions of different health service utilizations were 68.8, 14.7, and 16.5%. The different health service utilizations of UEBMI accounts for 71.4, 12.6, and 16.0%, respectively. Therefore, most middle-aged and older adults did not have any health service utilization.

**Table 3:** The proportion of different health service utilization in SBMI

	*n*(%)
*NCMS*	
Without any health service utilization	7869(72.3)
With only outpatient health service utilization	1655(15.2)
With inpatient health service utilization	1362(12.5)
*URBMI*	
Without any health service utilization	1098(68.8)
With only outpatient health service utilization	235(14.7)
With inpatient health service utilization	263(16.5)
*UEBMI*	
Without any health service utilization	432(71.4)
With only outpatient health service utilization	76(12.6)
With inpatient health service utilization	97(16.0)

Considering that the dependent variable in the group without health service utilization equals zero, our study performed linear quantile mixed regression on middle-aged and older adults with only outpatient health service utilization or inpatient health service utilization. For convenience in presentation, our study selects five representative quantiles, namely, 0.1, 0.25, 0.5, 0.75, and 0.9. The estimation results for the group with only outpatient health service utilization were shown in Table 4, a significant positive association between URBMI and outpatient health service utilization at all quantiles was observed. The UEBMI was also significantly positive associated with outpatient health service utilization at 0.75 (β = 0.609, *p* < 0.01) and 0.9 (β = 0.692, *p* < 0.01) quantiles, and the results were consistent with the results in Table 2.

**Table 4:** Estimation results of samples with only outpatient health service utilization

Variables	LMM	Quantile				
		0.1	0.25	0.5	0.75	0.9
*Insurances*						
URBMI	0.370***	0.475**	0.295*	0.431***	0.365*	0.545**
	(0.120)	(0.191)	(0.170)	(0.126)	(0.187)	(0.259)
UEBMI	0.300	0.352	0.343	0.319	0.609***	0.692***
	(0.180)	(0.265)	(0.255)	(0.213)	(0.151)	(0.237)
Constant	6.100***	6.264***	6.190***	6.110***	6.270***	6.574***
	(0.320)	(0.284)	(0.346)	(0.315)	(0.414)	(0.356)
Observations	1966	1966	1966	1966	1966	1966
AIC	7241	7931	7489	7356	7579	8236
Log Likelihood	− 3588	− 3933	− 3713	− 3596	− 3758	− 4086

Notes: (a) Standardize coefficients are reported; standard errors in parentheses

(b) Significance level: ***p < 0.01, **p < 0.05, *p < 0.1

(c) We further controlled demographic and other control variables. And the results are not reported in detail

The estimation results for the group with inpatient medical expenditure were provided in Table 5, showing that the URBMI was significantly positive associated with inpatient health service utilization at all quantiles. These results demonstrated that URBMI was significantly associated with an improvement in inpatient health service utilization of the middle-aged and older adults, but this gain in health service utilization was smaller at medium quantiles. And a significant positive association between UEBMI and inpatient health service utilization was observed at 0.1 (β = 0.559, *p* < 0.01), 0.25 (β = 0.420, *p* < 0.05), 0.5 (β = 0.352, *p* < 0.05), and 0.75 (β = 0.306, *p* < 0.05) quantiles. These results showed that UEBMI was associated with an improvement in inpatient health service utilization of the middle-aged and older adults at most quantiles, but the gain in inpatient health service utilization was smaller at higher quantiles.

**Table 5:** Estimation results of samples with inpatient health service utilization

Variables	LMM	Quantile				
		0.1	0.25	0.5	0.75	0.9
*Insurances*						
URBMI	0.620***	0.631**	0.665***	0.492***	0.601***	0.578***
	(0.110)	(0.149)	(0.102)	(0.088)	(0.109)	(0.117)
UEBMI	0.270*	0.559***	0.420**	0.352**	0.306**	0.063
	(0.140)	(0.132)	(0.165)	(0.164)	(0.148)	(0.209)
Constant	8.800***	8.327***	8.352***	8.839***	9.158***	9.308***
	(0.270)	(0.438)	(0.387)	(0.294)	(0.370)	(0.466)
Observations	1722	1722	1722	1722	1722	1722
AIC	5680	6348	5811	5623	5898	6382
Log Likelihood	− 2808	− 3142	− 2874	− 2780	− 2917	− 3159

Notes: (a) Standardize coefficients are reported; standard errors in parentheses

(b) Significance level: ***p < 0.01, **p < 0.05, *p < 0.1

(c) We further controlled demographic and other control variables. And the results are not reported in detail

## Discussion

The purpose of this study was to investigate the association between China’s SBMI schemes and health service utilization of middle-aged and older adults at different quantiles. We used linear quantile mixed regressions to provide a comprehensive understanding of the relationship between the SBMI and health service utilization. Our study yielded two key findings.

First, our study found that URBMI and UEBMI were significantly positive associated with health service utilization compared with NCMS at most quantiles. These results were consistent with those of existing literature about the impact of medical insurance [4, 34]. And it could be explained as follows: compared with URBMI and UEBMI, the deductible of NCMS is generally high and the budget is relatively limited and thus the coverage is typically shallow [12, 35, 36]. Therefore, middle-aged and older adults from rural areas experience a certain degree of inequity in the health service utilization compared with middle-aged and older adults from urban areas.

Second, the results of the quantile regression analyses demonstrated that the associations between the SBMI and health service utilization largely differed at different quantiles. URBMI had the most significant gain in health service utilization for middle-aged and older adults with lower health service utilization, and UEBMI had the most significant utility in health service utilization for middle-aged and older adults with middle-level health service utilization. A likely account for this result was that middle-aged and older adults with high health service utilization might still cannot afford the medical expenses after reimbursements since the reimbursement rates of UEBMI, URBMI, and NCMS were 72, 50, and 40%, respectively [12]. And the limited utility of UEBMI for middle-aged and older adults with high inpatient health service utilization also confirmed this result. Due to the high deductibles for outpatient services, UEBMI had a limited impact on middle-aged and older adults with lower outpatient health service utilization. In addition, our results showed that the SBMI system had limited impact on the utilization of health services by the middle-aged and older adults who were overburdened with medical care. Because of the low national average premium per capita (2842.4, 1628.4, and 408 yuan under UEBMI, URBMI, and NCMS, respectively), middle-aged and older adults with lower health service utilization were more likely to benefit from the SBMI. This finding is consistent with previous literature [37, 38]. Previous studies have also proven that the rich people were more likely to benefit from medical insurance [39], and NCMS increased the utilization of health services. However, it increased the burden of medical expenses for the poor [40]. Thus, health inequity persisted in the middle-aged and older adults with various levels of health service utilization in China.

The results of this study provide insights that can contribute to policy design. First, due to the low reimbursement rates, the association between health service utilization and URBMI/UEBMI was limited for the middle-aged and older adults with high health service utilization. Second, reimbursement rates could be set according to the levels of medical expenditure. Third, health inequity still exists, especially in urban and rural areas. Future healthcare reforms in China should not only focus on expanding coverage, but also on improving the equity of distribution of healthcare benefits [41], and the gaps in the benefits package across the SBMI systems should be further reduced [18].

Expanding health insurance coverage is a critical step toward health equity. Patients are supposed to be able to access and afford healthcare [42]. However, various programs of the SBMI have different effects on the utilization of medical services among the middle-aged and older adults in China. The underlying reason is the design of the benefits package of basic social health insurance. In the near future, the gap in the utilization of health services among the middle-aged and older adults will continue to exist and should be properly settled.

We also made several contributions to the existing literature. Our study can be regarded as a continuation of the health equity research in the process of SBMI coverage in China, which expands the application scope of health equity. Meanwhile, using a quantile regression approach, we further explored the impact of different SBMI programs on the utilization of health services for the middle-aged and older adults in China, which expands the application scope of quantile regression.

### Limitations

Our findings should be interpreted with caution because of the following limitations. First, we used self-reported survey data, which might suffer from measurement error. Second, our study only used medical expenditure to measure health service utilization, so the validity of measurement might be a little bit limited. Although we further controlled some variables, it could still be a potential problem. Third, the coefficients of UEBMI were smaller than URBMI, which might due to the fact that UEBMI beneficiaries were usually employed and younger and healthier than URBMI. It suggested that there might be some unobserved variables concerning differences in healthcare need that had not been included in this study. Fourth, our study only considered three SBMI programs in China and did not consider other commercial medical insurance systems, and it also ignored the middle-aged and older adults without the SBMI. Those samples can be further studied in future works.

## Conclusions

We used the CHARLS dataset to explore the relationship between SBMI and health service utilization for middle-aged and older adults in urban and rural Chinese areas. Our study suggests that while accelerating the promotion of SBMI coverage, policymakers should reduce the gaps in the benefits package across different schemes. Policy efforts should further focus on adjusting social health insurance and optimizing healthcare resource allocation in order to enhance the effective utilization of healthcare services and control the increase of costs among middle-aged and older adults [41]. Furthermore, inequity can be gradually reduced through continuous adjustment of the medical insurance scheme, thereby effectively targeting economically disadvantaged enrollees [43].

## Supplementary information

**Additional file 1: Table S1.** Descriptive statistics of Provinces and Chronic.

## Data Availability

The datasets supporting the conclusions of this article are publicly available on the CHARLS website http://charls.pku.edu.cn/en. The datasets we used in this study can be applied after logging on. Once approved, various waves of CHARLS could be downloaded at http://charls.pku.edu.cn/pages/data/111/en.html.

## Abbreviations

CHARLS: China health and retirement longitudinal study
NCMS: New cooperative medical scheme
SBMI: Social basic medical insurance
UEBMI: Urban employee basic medical insurance
URBMI: Urban resident basic medical insurance
