# Supplementary material for: Medical insurance and health equity in health service utilization among the middle-aged and older adults in China: a quantile regression approach
Source: BMC Health Serv Res. 2020 Jun 17;20:553. doi: 10.1186/s12913-020-05423-y (PMC7302153; doi:10.1186/s12913-020-05423-y)
Supplement: Supplementary file 1 — Additional file 1: Table S1. Descriptive statistics of Provinces and Chronic. [file 12913_2020_5423_MOESM1_ESM.docx]

Table S1 summarizes the descriptive statistics of Geographic location (Provinces) and Chronic condition (Chronic).

**Table S1.** Descriptive statistics of Provinces and Chronic

|  | n(%) |
| --- | --- |
| Provinces |  |
| Yunnan | 840(6.4) |
| Fujian | 382(2.9) |
| Qinghai | 130(1.0) |
| Sichuan | 1146(8.8) |
| Hebei | 596(4.6) |
| Jiangxi | 620(4.7) |
| Xinjiang | 63(0.5) |
| Beijing | 25(0.2) |
| Neimenggu | 583(4.5) |
| Jiangsu | 585(4.5) |
| 18 other provinces | 8117(62.0) |
| *Chronic* |  |
| Hypertension |  |
| Yes | 3241(24.8) |
| No | 9846(75.2) |
| Dyslipidemia |  |
| Yes | 1382(10.6) |
| No | 11705(89.4) |
| Diabetes or high blood sugar |  |
| Yes | 796(6.1) |
| No | 12291(93.9) |
| Cancer or malignant tumor |  |
| Yes | 144(1.1) |
| No | 12943(98.9) |
| Chronic lung diseases |  |
| Yes | 1519(11.6) |
| No | 11568(88.4) |
| Liver disease |  |
| Yes | 620(4.7) |
| No | 12467(95.3) |
| Heart attack |  |
| Yes | 1689(12.9) |
| No | 11398(87.1) |
| Stroke |  |
| Yes | 307(2.4) |
| No | 12780(97.6) |
| Kidney disease |  |
| Yes | 945(7.2) |
| No | 12142(92.8) |
| Stomach or other digestive disease |  |
| Yes | 3569(27.3) |
| No | 9518(72.7) |
| Emotional, nervous, or psychiatric problems |  |
| Yes | 181(1.4) |
| No | 12906(98.6) |
| Memory-related disease |  |
| Yes | 203(1.6) |
| No | 12884(98.4) |
| Arthritis or rheumatism |  |
| Yes | 5095(38.9) |
| No | 7992(61.1) |
| Asthma |  |
| Yes | 579(4.4) |
| No | 12508(95.6) |
